# Supplementary material for: Optimization design of centrifugal impeller based on Bezier surface and FFD space grid parameterization
Source: PLoS One. 2024 Nov 20;19(11):e0310792. doi: 10.1371/journal.pone.0310792 (PMC11578478; doi:10.1371/journal.pone.0310792)
Supplement: Attachment — Submitted filename: Response to Reviewers.docx [file pone.0310792.s001.docx]

### Revision Report

**Title:** Optimization Design of Centrifugal Impeller Based on Bezier surface and FFD Space Grid Parameterization

**PONE-D-24-17203**

Dear editor and reviewer,

We would like to thank you very much for the valuable comments from the reviewers. The comments have been very helpful in improving our manuscript. We have attempted to address all the comments and suggestions in the revision, polish the language, as well as improve several pictures quality. The following table presents the point-to-point highlights for our revised version.

### Responses to Journal

| **No.** | **Comments and our responses** |
| --- | --- |
| **1)** | **Comments：**  When submitting your revision, we need you to address these additional requirements.  Please ensure that your manuscript meets PLOS ONE's style requirements, including those for file naming. The PLOS ONE style templates can be found at  [https://journals.plos.org/plosone/s/file?id=wjVg/PLOSOne formatting_sample_main_body.pdf](https://journals.plos.org/plosone/s/file?id=wjVg/PLOSOne%20formatting_sample_main_body.pdf)  <https://journals.plos.org/plosone/s/file?id=wjVg/PLOSOne_formatting_sample_main_body.pdf>  https://journals.plos.org/plosone/s/file?id=ba62/PLOSOne_formatting_sample_title_authors_affiliations.pdf  **Responses**:  Thank you for the comments.  We have ensured that our manuscript now fully adheres to PLOS ONE’s style requirements, including the correct file naming conventions. We have used the provided PLOS ONE style templates to format the main body, title page, and author affiliations appropriately. |
| **2)** | **Comments：**  We note that your Data Availability Statement is currently as follows: "All relevant data are within the manuscript and its Supporting Information files."  Please confirm at this time whether or not your submission contains all raw data required to replicate the results of your study. Authors must share the "minimal data set" for their submission. PLOS defines the minimal data set to consist of the data required to replicate all study findings reported in the article, as well as related metadata and methods (https://journals.plos.org/plosone/s/data-availability#loc-minimal-data-set-definition).  For example, authors should submit the following data:  - The values behind the means, standard deviations and other measures reported;  - The values used to build graphs;  - The points extracted from images for analysis.  Authors do not need to submit their entire data set if only a portion of the data was used in the reported study. If your submission does not contain these data, please either upload them as Supporting Information files or deposit them to a stable, public repository and provide us with the relevant URLs, DOIs, or accession numbers. For a list of recommended repositories, please see https://journals.plos.org/plosone/ s/recommended-repositories. If there are ethical or legal restrictions on sharing a de-identified data set, please explain them in detail (e.g., data contain potentially sensitive information, data are owned by a third-party organization, etc.) and who has imposed them (e.g., an ethics committee). Please also provide contact information for a data access committee, ethics committee, or other institutional body to which data requests may be sent. If data are owned by a third party, please indicate how others may request data access.  **Responses**:  Thank you for the comments.  We confirm that our submission contains all the raw data required to replicate the results of our study. These data are included within the manuscript and its Supporting Information files. |

### Responses to Reviewer #1

| **No.** | **Comments and our responses** |
| --- | --- |
| **1)** | **Comments：**  The research paper titled "Optimization Design of Centrifugal Impeller Based on Bezier Surface and FFD Space Grid Parameterization" introduces a cutting-edge optimization design approach aimed at overcoming challenges associated with a high number of design variables, rigid configurations, and low optimization efficiency.  The utilization of a Bezier surface and Free-Form Deformation (FFD) model plays a crucial role in enhancing the design process. The FFD model was chosen for its ability to provide flexibility in manipulating the geometry of the impeller, allowing for efficient optimization and improved performance.  The introduction section of the paper can be enhanced by incorporating recent studies such as "CFD Analysis of a Vertical Axis Wind Turbine" (Mathematical Modelling of Fluid Dynamics and Nanofluids) to provide a solid grounding for the research context.  Comparative analysis with existing published works is essential to demonstrate the significance of the proposed methodology. By juxtaposing the results obtained in this study with those in the literature, a comprehensive evaluation can be made to showcase the advancements and contributions of the research.  The novelty of this work lies in the integration of Bezier surface and FFD model for optimizing the design of centrifugal impellers. This innovative approach offers a unique solution to the challenges faced in traditional design processes, leading to enhanced efficiency and performance.  In conclusion, the key findings of this research can be summarized into clear and impactful points highlighting the successful application of the proposed methodology, the improvements achieved in design efficiency, and the potential for broader implications in the field of impeller design. By emphasizing these key takeaways, the conclusion can effectively underscore the significance and implications of the study.  **Responses**:  We truly appreciate this comment.  The proposed method combines the strengths of surface and spatial mesh parameterization to develop both the Bezier surface model and the free-form deformation (FFD) model. This approach enables flexible and precise reshaping of centrifugal compressor blades while significantly reducing the number of global design variables required for rapid optimization.  To strengthen the research background, we have incorporated several recent studies into the introduction and updated older references to those published within the last five years. Please refer to the text for signs of modification  This paper introduces, for the first time, the combined use of Bezier surface parameterization and FFD parameterization methods to optimize the design of centrifugal impellers. This approach can efficiently and rapidly enhance the overall aerodynamic performance of centrifugal pressurized impellers, offering significant improvements compared to using a single parameterization method. |

### Responses to Reviewer #2

| **No.** | **Comments and our responses** |
| --- | --- |
| **1)** | **Comments：**  The over all it is a good study. But however, the presentation of the results should be improved. The quality of the graphs is not good. The language of article is also very weak it should be improved.  **Responses**:  We truly appreciate this comment.  Thank you for your constructive feedback. We have addressed your concerns by improving the quality of the graphs to ensure they are clearer and more informative. Additionally, we have carefully reviewed and refined the language throughout the manuscript to enhance its readability and clarity. We believe these revisions significantly improve the presentation of our results and the overall quality of the paper. Please refer to the Revised Manuscript with Track Changes. |

Dear Editor:

Thank you once again for the thorough review and valuable feedback provided by the reviewers on our manuscript submitted to your esteemed journal. We greatly appreciate the constructive criticisms and concerns raised.

In response to their insightful suggestions, we have carefully reviewed and made comprehensive revisions to the manuscript. We fully understand the issues highlighted and have provided detailed explanations and amendments for each concern. We believe these revisions significantly enhance the quality of the paper and further strengthen its academic value and reliability.

We are committed to continuing our collaboration with the journal to refine the manuscript, ensuring it meets the highest standards. We will actively incorporate the reviewers' recommendations and strive to present a final version that fully aligns with the journal's requirements. We would like to express our gratitude once again for the attention and support given to our research by you and the journal. We hope to share this research outcome with the scientific community and contribute to the advancement of the field. Should there be any further questions or guidance, we would be more than willing to cooperate and respond promptly.

Thank you again for your valuable time and dedication.

Sincerely
